# Transcriptome profiling of gene expression during immunisation trial against *Fasciola hepatica*: identification of genes and pathways involved in conferring immunoprotection in a murine model

**DOI:** 10.1186/s12879-017-2205-3

**Published:** 2017-01-23

**Authors:** Jose Rojas-Caraballo, Julio López-Abán, Darwin Andrés Moreno-Pérez, Belén Vicente, Pedro Fernández-Soto, Esther del Olmo, Manuel Alfonso Patarroyo, Antonio Muro

**Affiliations:** 1 Parasite and Molecular Immunology Laboratory, Tropical Disease Research Centre, (IBSAL-CIETUS), University of Salamanca, Salamanca, Spain; 2 Molecular Biology and Immunology Department, Fundación Instituto de Inmunología de Colombia (FIDIC), Bogotá, Colombia; 3 Basic Sciences Department, School of Medicine and Health Sciences, Universidad del Rosario, Bogotá, Colombia; 4 Pharmaceutical Chemistry Department, (IBSAL-CIETUS), University of Salamanca, Salamanca, Spain; 5 Present address: Centro de Investigación en Salud para el Trópico (CIST), Facultad de Medicina, Universidad Cooperativa de Colombia, Santa Marta, Magdalena Colombia

**Keywords:** Fasciolosis, Vaccine, Epitope, Immunology, Microarrays, Gene expression

## Abstract

**Background:**

Fasciolosis remains a significant food-borne trematode disease causing high morbidity around the world and affecting grazing animals and humans. A deeper understanding concerning the molecular mechanisms by which *Fasciola hepatica* infection occurs, as well as the molecular basis involved in acquiring protection is extremely important when designing and selecting new vaccine candidates. The present study provides a first report of microarray-based technology for describing changes in the splenic gene expression profile for mice immunised with a highly effective, protection-inducing, multi-epitope, subunit-based, chemically-synthesised vaccine candidate against *F. hepatica*.

**Methods:**

The mice were immunised with synthetic peptides containing B- and T-cell epitopes, which are derived from *F. hepatica* cathepsin B and amoebapore proteins, as novel vaccine candidates against *F. hepatica* formulated in an adjuvant adaptation vaccination system; they were experimentally challenged with *F. hepatica* metacercariae. Spleen RNA from mice immunised with the highest protection-inducing synthetic peptides was isolated, amplified and labelled using Affymetrix standardised protocols. Data was then background corrected, normalised and the expression signal was calculated. The Ingenuity Pathway Analysis tool was then used for analysing differentially expressed gene identifiers for annotating bio-functions and constructing and visualising molecular interaction networks.

**Results:**

Mice immunised with a combination of three peptides containing T-cell epitopes induced high protection against experimental challenge according to survival rates and hepatic damage scores. It also induced differential expression of 820 genes, 168 genes being up-regulated and 652 genes being down-regulated, *p* value <0.05, fold change ranging from −2.944 to 7.632. A functional study of these genes revealed changes in the pathways related to nitric oxide and reactive oxygen species production, Interleukin-12 signalling and production in macrophages and Interleukin-8 signalling with up-regulation of S100 calcium-binding protein A8, Matrix metallopeptidase 9 and CXC chemokine receptor 2 genes.

**Conclusion:**

The data obtained in the present study provided us with a more comprehensive overview concerning the possible molecular pathways implied in inducing protection against *F. hepatica* in a murine model, which could be useful for evaluating future vaccine candidates.

**Electronic supplementary material:**

The online version of this article (doi:10.1186/s12879-017-2205-3) contains supplementary material, which is available to authorized users.

## Background

Fasciolosis is one of the most widespread food-borne trematode diseases around the world, causing significant economic losses in developing countries (estimated at over 3 billion dollars per year) and affecting a wide range of mammals, mainly ruminants [1, 2]. *F. hepatica* also causes disease in human beings, most cases being reported in Andean countries, Egypt and Iran [3–5]. Several reports have estimated that up to 17 million people are infected and 91 to 170 million people live in areas having a high risk of acquiring the disease [6].

Chronic *F. hepatica* infection causes hepatomegaly, gallbladder and biliary duct thickening and dilatation, leading to cholangitis, cholecystitis, usually accompanied by obstruction of the biliary ducts. *F. hepatica* infection also causes hepatic tissue damage and parenchymal destruction by juvenile fluke migration until the biliary ducts are reached [7]. The immune response induced by *F. hepatica* infection has been well described in experimental models, typically being characterised by the presence of a dominant Th2 response with IL-4, IL-5 and IL-13 secretion by spleen cells and regulatory cytokines IL-10 and TGF-β by macrophages and dendritic cells and Th1 immune response suppression in murine models [8–10]. A dominant Th2 immune response also occurs during the chronic phase of the disease in cattle, involving high levels of IgG1 and little or no IgG2a; it has also been shown that susceptibility to infection is correlated with the IgG1/IgG2a ratio, as well as IL-4/IFN-γ levels [11–14]. Such immunomodulation/immunoregulation of the host’s immune response by helminthic parasites is a key factor for successful infection and parasite survival within host tissues. Besides the large body of knowledge concerning an immune response in *F. hepatica* infection, the precise molecular mechanisms leading to protection are not yet well understood.

Fasciolosis control in animals and humans is currently based on the mass administration of triclabendazole which is active against both adult parasites located in the bile ducts and immature flukes migrating through the liver. This strategy has led to progressive drug-resistance in animals [15–17]. Despite there is evidence that a number of drugs have activity against *F. hepatica*, it seems to be only partial, acting only either on the immature or mature flukes. Also, the potential risk of secondary effects makes the replacement of triclabendazole an ineffective strategy for its control [18–20]. Developing an effective vaccine represents one of the most appealing strategies for preventing this disease and reducing the risk of infection in humans. No commercial vaccines are yet available for *F. hepatica* although significant progress has been made regarding attempts at developing effective vaccines against infection caused by other parasites, only a few vaccines have been successfully tested against helminthic infection in animals [21–24]. Several antigens have been identified and tested as vaccine candidates against *F. hepatica* infection. Most studies have focused on using cathepsins, leucine-aminopeptidase, haemoglobin, fatty-acid binding proteins and glutathione S-transferase as vaccine candidates. Such antigens have mainly been produced as recombinant proteins and administered in different adjuvant-type formulations. Vaccine efficacy has ranged from 13 to 97% in cattle, measured as liver-fluke burden reduction [25, 26]. Subunit-based, chemically-synthesised vaccine candidates represent a promising vaccine development strategy. Using synthetic peptides offers a wide range of advantages: inexpensive production, induction of a strong immune response, avoid using living organisms thus minimising the risk of acquiring disease, sequences which can interfere with vaccination success become eliminated and peptides from different antigens can be conjugated to the same carrier [27, 28].

A previous study by our group involving the use of bioinformatics tools showed the protection-inducing ability of *F. hepatica* cathepsin B and amoebapore-derived B- and T-cell epitopes which had been chemically-synthesised and selected as vaccine candidates according to their induced immune response in a murine model, reaching up to 66.7% protection after immunisation [29]. According to the results obtained in the above mentioned article, where we found reduction in hepatic damage and increase of survival rates, in the present study we hypothesised that a combination of peptides with different induced-immune responses could act in a synergic way in order to improve the immunoprotection in mice. In the present study we used the so-called ADAD vaccination system. Previous reports by our group have demonstrated the improvement in immunoprotection trials against *F. hepatica* when using this vaccination system [30].

Better understanding concerning the molecular mechanisms by which *F. hepatica* infection occurs, as well as the molecular basis involved in obtaining protection, is extremely important in designing and selecting new vaccine candidates. The present study involved using a microarray-based methodology for studying the gene expression profile in the spleen of mice immunised with a highly protective anti-*F. hepatica* vaccine candidate using a combination of three synthetic peptides containing T-cell epitopes, derived from *F. hepatica* cathepsin B and amoebapore proteins, and the possible molecular pathways involved in inducing protection.

## Methods

### Ethical statement

Animal procedures complied with Spanish (Real Decreto RD53/2013) and European Union (European Directive 2010/63/CE) guidelines regarding animal experimentation for the protection and humane use of laboratory animals. The University of Salamanca’s accredited Animal Experimentation Facilities (Registration number: PAE/SA/001) were used for such procedures. The University of Salamanca’s Ethics Committee also approved the procedures used in this study (Permit Number: 8402). All efforts were made to minimise animal suffering.

### Animals and parasites

Female, 6 week-old CD1 and BALB/c mice (Charles River Laboratories, Barcelona, Spain) weighing 20 to 35 g were used for the experiments. The animals were maintained in the University of Salamanca’s animal care facility and kept in plastic boxes with food and water *ad libitum* *.* The animals were maintained with regular 12 h light–dark periods at 20 °C. *F. hepatica* metacercariae were provided by Ridgeway Research Ltd (Gloucestershire, UK) and stored at 4 °C on 0.4% carboxymethylcellulose until use. Metacercariae viability was confirmed by microscope observation before infection.

### In vivo protection studies: antigens, vaccine formulation and immunisation trials

A previous study by our group led to identifying seven *F. hepatica* cathepsin B and amoebapore-derived peptides containing B- and T-cell epitopes as promising vaccine candidates [29]. Briefly, from partial publically available information in databases concerning *F. hepatica* protein amino acid sequences, target-proteins were selected in line with the following criteria: proteins having a signal peptide sequence as predicted by SignalP 3.0 server, freely available at http://www.cbs.dtu.dk/services/SignalP-3.0/ [31] and proteins lacking a trans-membrane domain as predicted by the TMHMM v2.0 server. Only proteins having both a signal peptide and no trans-membrane domains were selected and grouped into common families. ClustalW was used for the multiple alignment of target proteins’ amino acid sequences and only conserved or semi-conserved regions were chosen for B- and T-cell epitope prediction [32]. Subsequently, B- and T-cell epitopes were chemically-synthesised and used for inoculating BALB/c mice using an adjuvant adaptation (ADAD) vaccination system [30]. Each peptide’s induced immune response and protection-inducing ability was assessed in in vivo studies. Seven peptides containing B- or T-cell epitopes derived from *F. hepatica* cathepsin B and amoebapore proteins were identified as promising vaccine candidates.

Such single peptides were combined in the present study, according to the induced immune response and type of epitope. The amino acid sequence of each peptide used here, as well as information concerning the protein it came from is given (Additional file 1: Table S1A).

Forty-nine CD1 mice were divided into 7 groups (7 mice each) as follows: group 1 consisted of untreated and uninfected controls, group 2 untreated and infected controls, group 3 adjuvant-administered and infected controls, group 4 those immunised with the combination of peptides B6 and T14, group 5 mice immunised with peptides B1, B5 and B6, group 6 mice immunised with peptides T14, T15 and T16 and group 7 was immunised with peptides containing B- and T-cell epitopes (B1, B2, B5, B6, T14, T15 and T16). The mice were subcutaneously immunised using an ADAD system [30]. Briefly, the ADAD vaccination system included the vaccine antigen, an immunomodulator (natural or chemically-synthesised), together with non-haemolytic adjuvant *Quillaja saponaria* saponins to form an emulsion with non-mineral oil in a 70/30 oil/water ratio. Vaccination with this system included a set of 2 subcutaneous injections. The first (also called adaptation) contained *Q. saponaria* and the immunomodulator emulsified in non-mineral oil, but without the vaccine antigen; the second injection was administered 5 days after adaptation and contained the vaccine antigen with *Q. saponaria* and the immunodulator in the emulsion oil. Individual mouse immunisation involved injecting doses formulated as follows: 100 μg chemically synthesised aliphatic diamine immunomodulator AA0029 [33] together with 20 μg *Q. saponaria* and, when evaluated, 10 μg of each peptide. A final 100 μL/injection volume was emulsified with non-mineral oil (Montanide ISA763A, SEPPIC, Paris, France) in a 70/30 oil/water ratio. Mice were immunised on day 0 and two booster doses with 100 μL of the aforementioned preparations were administered on days 14 and 28.

### Experimental infection and protection assessment

Animals included in this study (except non-infected controls – Group 1) were orally challenged with 7 *F. hepatica* metacercariae 2 weeks after the last immunisation. Humane endpoints were applied when an evidence of severe pain, excessive distress, suffering or an impending death was observable in any of the animals and then euthanised with an intraperitoneal injection of 100 mg/kg pentobarbital and necropsied to score hepatic damage. Day 42 post-infection (p.i) was set as the trial’s end-point; protected mice were considered to be animals remaining alive until day 42 p.i. Mice still remaining alive were then humanely euthanised and necropsied as above. Synthetic peptides’ protection-inducing ability was then calculated on the basis of survival rates, according to Kaplan Meier estimators [34]. All animals were necropsied to score hepatic damage. Two experienced pathologists independently evaluated liver lesions without knowing which group the livers belonged to. Changes regarding size, colour and consistency concerning blood vessels, bile ducts and surface wounds were evaluated; a score was assigned to each feature: 0 points if no lesion was observed, 1 point if a liver lobe was affected, 2 if an entire lobe was affected and 3 if more than 1 lobe was affected. No lesion was assigned when the sum was 0 points, mild (+) for 1–5 points, moderate (++) for 6–10 points and severe (+++) for 11–14 points [35]. The infected mouse survival rate percentage was then calculated as the ratio of the number of surviving experimental mice on day 42 p.i and the total of experimental mice in each group.

### Measuring antibody response

Sera from mice immunised with the aforementioned formulations were analysed by ELISA for measuring total IgG levels. The presence of specific antibodies raised against *F. hepatica* was also detected in all the mice infected with *F. hepatica* metacercariae according to the methodology previously described [29].

### Selecting the most protective antigen combination and immunisation trial

Following in vivo protection studies in mice, the combination of peptides inducing the highest level of protection was selected for gene expression profile analysis, taking survival rates and hepatic damage score into account. Six female BALB/c mice were included in this study and divided into 2 groups (3 mice each) for the immunisation trial. Mice in group 8 (*n* = 3) received no treatment throughout (i.e. regarding their immunisation schedule). Mice in group 9 (*n* = 3) were subcutaneously immunised with 10 μg of antigen, 100 μg AA0029 and 20 μg *Q. saponaria* emulsified with Montanide ISA763A on day 0. Two booster doses with 100 μL of the aforementioned preparations were injected on days 14 and 28. The mice were humanely euthanised by intraperitoneal injection of pentobarbital (100 mg/kg) two weeks after the last immunisation and necropsied for spleen recovery and RNA isolation. Blood samples were also taken prior to each vaccination dose and at the time of necropsy.

### Spleen RNA isolation

An RNeasy Mini Kit (Qiagen, Valencia, CA, USA) was used for isolating total RNA from splenocytes from both untreated and immunised mice, according to the manufacturer’s instructions. A Nanodrop-1000 (Nanodrop Technologies, Wilmington, USA) was used for measuring the total amount of RNA and the Agilent 2100 Bioanalyzer platform was used for ascertaining quality. Only RNA samples having an RNA integrity number (RIN) between 8 and 10 (on a 1 to 10 scale, 1 being the lowest quality and 10 the highest) were used for the next procedures. Six individual RNA samples were thus used for cDNA synthesis and microarray analysis.

### Microarray processing

A Mouse Gene 1.0 ST Affymetrix microarray was used in this study. Labelling and microarray hybridisation were carried out according to previously established Affymetrix protocols. The approach involved synthesising first and second strand cDNA, double-stranded cDNA purification, cRNA synthesis by in vitro transcription, biotin-labelled cRNA recovery and quantitation, cRNA fragmentation and subsequent hybridisation to microarray slide and post-hybridisation washing. A streptavidin-coupled fluorescent dye was used for detecting hybridised cRNA. Briefly, 100–300 ng total RNA was amplified and labelled using an Ambion WT expression kit (Ambion, Santa Clara, CA, USA) and then hybridised to mouse gene 1.0 ST Array (Affymetrix). The Affymetrix GeneChip system was used for washing and scanning (GeneChip Hybridization Oven 640, GeneChip Fluidics Station 450 and GeneChip Scanner 7G). The 169-format GeneChip Mouse Gene 1.0 ST Array contained around 25 probes designed along the full-length of 28,853 well-annotated genes for mice. The array was designed on the mouse genome sequence (UCSC mm8, NCBI build 36) with comprehensive coverage of RefSeq, putative complete CDS GenBank transcripts, all Ensembl transcript classes and syntenically mapped full-length human and rat mRNAs and RefSeq NMs. The GeneChip Mouse Gene 1.0 ST Array covers 100% of the NM sequences in the RefSeq database (http://www.affymetrix.com/support/index.affx).

### Microarray data analysis: normalisation, signal calculation, differential expression and functional profiling

Cell intensity files (.CEL) were created using established Affymetrix microarray analysis parameters. The robust microarray analysis algorithm was used for background correction, intra- and inter-microarray normalisation and expression signal calculations [36–38]. Once the absolute expression signal for each gene (i.e. the signal value for each probe set) had been calculated in each microarray, a method called significance analysis of microarray was used for calculating significant differential expression and finding the gene probe sets characterising highly metastatic samples [39]. This method involves permutations providing robust statistical inference of the most significant genes and, by using a false discovery rate (FDR), adjusting the raw *p*-values to take multiple testing into account [40]. A <0.05 FDR cut-off was used for all differential expression calculations. The Ingenuity Pathway Analysis (IPA) tool (Ingenuity Systems, Mountain view, CA, USA) was used for identifying the most significant biological mechanisms, pathways and functional categories and also to construct and visualise molecular interaction networks.

### PCR for experimental microarray validation

RNA from each biological sample was reserved for carrying out a semi-quantitative reverse-transcription PCR to validate a subset of the microarray data. Equal amounts of total RNA from each experimental group were pooled and reverse transcribed using a 1st strand cDNA synthesis kit for reverse-transcription-PCR (AMV) (Roche Applied Sciences, Indianapolis, IN, USA), 20 μL final volume, according to the manufacturer’s instructions. The cDNA concentration obtained was measured using a Nanodrop-1000 reader (Nanodrop Technologies, Wilmington, DE, USA). Genes and primers used for PCR (Additional file 1: Table S2B) were representative of the transcripts which were significantly up- or down-regulated during microarray analysis and designed using Primer3 software, freely available at http://bioinfo.ut.ee/primer3-0.4.0/. Primers were designed to be specific for each up- and down-regulated gene so selected and ensure that PCR products would be 140–280 bp. Twenty-five PCR amplification cycles were performed on a Mastercycler gradient (Eppendorf) thermocycler, as follows: denaturing at 94 °C for 30 s, annealing at 55 °C for 30 s (depending on Tm for each primer) and a final extension step at 72 °C for 5 min. PCR products were visualised on 2.0% (wt/vol) agarose gels with ethidium bromide. Genomic DNA contamination was ruled out for each RNA sample. Free ImageJ software (https://imagej.nih.gov/ij/) [41] was used for the quantitative digital analysis of the image data from electrophoresis gels.

### Statistical analysis

The results were expressed as the mean and standard error of the mean. Normal data distribution was checked using a non-parametric Kolmogorov-Smirnov test. Differences between groups were found using a one-way ANOVA test and Tukey’s honest significance test or the Kruskal Wallis test. Statistical analysis was considered significant at *p* < 0.05. SPSS 21.0 was used for graphical representation. The R statistical software was used for bioinformatics analysis, using Bioconductor [42] and GATExplorer [43] custom packages. Kaplan-Meier survival curves were used for evaluating survival rates.

## Results

### Immunisation and experimental infection

An ELISA assay was performed during two stages of our experimental procedure to assess the success of the immunisation trial and infection. Each group of mice immunised with any of the synthetic-peptide combinations induced levels of IgG. It was observed that the highest levels of IgG were induced in the group of mice immunised with the synthetic peptides containing B-cell epitopes. High levels of IgG raised against the excretory/secretory antigen from *F. hepatica* in orally infected mice were also detected. No IgG levels were detected in any of mice belonging to the non-immunised-uninfected control group (Group 1) (Additional file 2: Figure S1).

### In vivo protection studies: measuring the survival rates

**Fig. 1:**
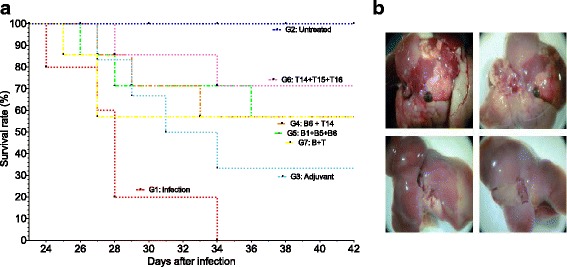
Immunoprotection in mice immunised with the synthetic peptides. **a** Kaplan-Meier curves depicting survival rates in mice immunised with any combination of peptides containing B- and T-cell epitopes as vaccine candidates which had also been orally infected with *F. hepatica* metacercariae. The survival rates of mice from both untreated and infected controls are also represented. The humane endpoint was established when an indicator of severe pain, excessive distress, suffering or an impending death was observed in any animal which was then euthanised with an intraperitoneal injection of pentobarbital at 100 mg/kg using 30 g needles. **b** Representative macroscopic lesion in infected mice (group 1, *upper part*) compared to mice immunised with a combination of peptides containing T-cell epitopes (T14 + T15 + T16; group 6, *lower part*)

Immunising mice with any combination of synthetic peptides led to enhancing their survival rates compared to the non-immunised and infected control group. Mice from the non-immunised and infected control group died between days 24 and 34 p. i. Taking this issue into account, protected mice were considered to be animals remaining alive until day 42 p.i; on the contrary, non-protected mice were considered animals which died before day 42 p.i. Figure 1a shows that immunising mice with any combination of the synthetic peptides containing either B- or T-cell epitopes induced some degree of protection against experimental infection with *F. hepatica* metacercariae.

A 71.4% (5 of 7 mice alive) survival rate was obtained in the group of mice immunised with the combination of peptides containing T-cell epitopes (T14, T15 and T16; Group 6), thus representing the highest survival rate obtained in the present study (Fig. 1a). All mice from group g4 (immunised with peptides B6 + T14), g5 (immunised with peptides B1 + B5 + B6) and g7 (immunised with peptides B1 + B2 + B5 + B6 + T14 + T15 + T16) achieved a 57.1% survival rate (4 of 7 mice alive). However, protection-inducing ability was ranked as follows: (B1 + B5 + B6; Group 5) > (B6 + T14; Group 4) > (B1 + B2 + B5 + B6 + T14 + T15 + T16; Group 7), taking the time of each animal’s death into account in the experimental groups (Fig. 1a). Mice died in the following days: group 2 on days 24 (1 mouse), 27 (2 mice), 28 (3 mice) and 34 (1 mouse); group 3 on days 27, 29, 31 and 34 (1 mouse each day); group 4 on days 27, 29 and 33 (1 mouse each day); group 5 on days 26, 28 and 36 (1 mouse each day); group 6 on days 28 and 34 (1 mouse each day) and group 7 on days 25 (1 mouse) and 27 (2 mice).

### Hepatic lesion scores

Forty-two days after experimental infection, all surviving mice were humanely euthanised and necropsied for evaluating hepatic lesions. This revealed that protected mice had lower hepatic damage scores than non-protected mice ([6.3 ± 0.5] *cf* [12.2 ± 0.4]) (*p* < 0.05). Mice having minor lesions were also classified (0 to 5 score), as were moderate lesions (6 to 10 score) and severe lesions (11 to 14 score). Non-protected mice only had moderate (30%) or severe lesions (70%) whilst protected-mice mainly had minor (48%) or moderate lesions (38%), thereby indicating a correlation between protection and hepatic damage score.

The group of mice immunised with the combination of peptides having T-cell epitopes (group 6) had the lowest hepatic damage score compared to the infected group ([6.6 ± 0.6] *cf* [12.0 ± 1.5]) (*p* < 0.05); a 45% reduction was observed in this group, again showing these peptides’ effectiveness as anti-*F. hepatica* infection vaccine candidates. Mice immunised with the full combination of peptides containing B- and T-cell epitopes (group 7) also had significantly reduced hepatic damage ([7.3 ± 1.3] *cf* [12.0 ± 1.5]) (*p* < 0.05), reaching 39% reduction compared to the untreated and infected control group. Table 1 gives the hepatic damage score for each group immunised with any combination of peptides used in the present study. There was some reduction, non significant (22%) ([9.3 ± 1.8] *cf* [12.0 ± 1.5]) (*p* > 0.05) regarding liver damage in the group 3 (mice immunised with ADAD components). A representative image of macroscopic lesion in the liver of mice infected (group 1) and mice immunised with the combination of synthetic peptides containing T-cell epitopes (group 6) is shown (Fig. 1b).

**Table 1:** Assessing macroscopic hepatic lesions in CD1 mice

Group	Treatment	Lesion score (mean ± SEM)	Reduction (%)
1	Untreated uninfected		
2	Infected	12.0 ± 1.5	
3	Adjuvant treated	9.3 ± 1.8	22
4	B6 + T14	8.3 ± 1.8	31
5	B1 + B5 + B6	10.3 ± 1.6	14
6	T14 + T15 + T16	6.6 ± 0.6	45*
7	B1 + B2 + B5 + B6 + T14 + T15 + T16	7.3 ± 1.3	39*

The mice were immunised with combinations of synthetic peptides containing B- and T-cell epitopes using the ADAD vaccination system which had been orally challenged with 7 *F. hepatica* metacercariae

*p < 0.05 compared to infected controls

**Fig. 2:**
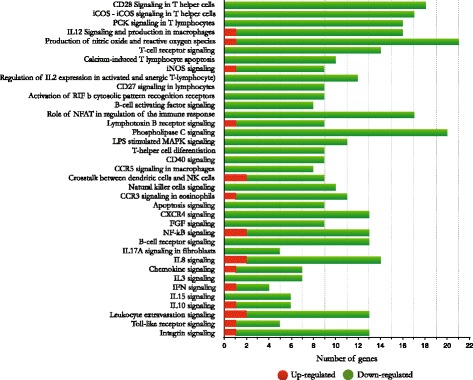
Ingenuity Pathway Analysis (IPA) showing the most representative canonical pathways associated with immune responses in the spleen of mice immunised with a combination of peptides containing T-cell epitopes (T14, T15 and T16). The figure shows the number of genes being differentially expressed compared to the untreated control group. *Red* represents the number of up-regulated genes whilst *green* represents down-regulated ones

The establishment of criteria for protection due to *F. hepatica* infection is well defined in natural infection hosts such as cows, lambs, goats and sheep. However, when using mouse as experimental model such criteria become unsuitable, mainly due to the very reduced worm recovered in the liver, hampering any statistical support. Thus, in the present study we did not take into account the number of worms recovered in the liver as a criterion for protection.

### Selecting the most protective antigen combination, immunisation trial and spleen RNA isolation

Following in vivo protection studies, the combination of peptides containing T-cell epitopes as the most promising vaccine candidate against *F. hepatica* infection was selected for the gene expression profile studies. Two-weeks after the last immunisation, the spleens from each mouse (6 in total) were aseptically removed and RNA was isolated. An Agilent 2100 Bioanalyzer was then used for assessing RNA integrity and purity. All RNA-isolated samples had an RNA integrity number of 10 (on a 1 to 10 scale, 1 being the lowest quality and 10 the highest), thus indicating an optimal starting product for cDNA synthesis and microarray design (Additional file 3: Figure S2).

### Microarray analysis and data pre-processing

The data discussed in this publication have been deposited in NCBI’s Gene Expression Omnibus [44] and is accessible through GEO Series accession number GSE69611 (https://www.ncbi.nlm.nih.gov/geo/query/acc.cgi?acc=GSE69611). The gene expression profile in the spleen of mice immunised with the combination of synthetic peptides containing T-cell epitopes (T14, T15 and T16) formulated in ADAD (Group 9) was studied as it had the highest immunoprotection rates in mice experimentally challenged with *F. hepatica* metacercariae compared to untreated mice (group 8), thereby providing insights into the potential pathways involved in inducing protection.

Raw data was quality assessed to ensure its integrity before any analysis was performed. Microarray data was background corrected by using the robust microarray analysis algorithm to eliminate background noise. A box plot showing the normalised unscaled standard error for each microarray is depicted (Additional file 4: Figure S3). Normalisation of data and subsequent differential expression signal calculation led to identifying 820 genes being differentially expressed (*p* < 0.05) as a result of immunisation with our vaccine candidate (peptides containing T-cell epitopes derived from *F. hepatica* cathepsin B and amoebapore proteins; comparison between groups 8 and 9). Signal intensity calculation for differentially expressed genes (fold change) from both down- and up-regulated genes ranged from −2.944 to 7.632. Additional file 5: Table S2, gives the complete list and provides additional information regarding each gene being differentially expressed. Immunising mice with our vaccine candidate led to the differential expression of 820 genes; 652 genes were down-regulated and 168 up-regulated. However, only 12 down-regulated genes (1.8%) had a fold change value lower than −2, whilst 37 up-regulated genes (22.0%) had a fold change value higher than 2.

### Pathway analysis

The free version of the IPA tool was used for data interpretation and comparing the non-immunised control group (group 8) to the group immunised with our vaccine candidate (group 9); 121 canonical pathways were identified having a *p*-value <0.05, 37 of them being associated with an immune response. Figure 2 shows the most representative immune response-associated pathways: CD28 signalling in T-helper cells, iCOS – iCOS signalling in T-helper cells, PKC signalling in T-lymphocytes, IL-12 signalling and production in macrophages and the production of nitric oxide and reactive oxygen species were the most significant pathways undergoing changes during our immunisation procedure. Strong down-regulation occurred with 391 (95.6%) genes being down-regulated but only 18 (4.4%) being up-regulated. Table 2 lists the genes having the highest differential expression and their participation in each canonical immune response associated pathway. Additional file 5: Table S3, provides a detailed list including all the signalling pathways and their associated-genes being differentially expressed by immunisation.

**Table 2:** Genes having the highest differential expression associated with immune response-related pathways

Gene symbol	Description	*p*-value	Fold change	Associated pathway
S100A8	S100 calcium binding protein A8	1.62E-03	5.934	IL12 signalling and production in macrophages
				Production of nitric oxide and reactive oxygen species
MMP9	Matrix metallopeptidase 9	3.07E-03	4.501	IL8 signalling
				Leukocyte extravasation signalling
CXCR2	Chemokine (C-X-C motif) receptor 2	3.39E-04	3.401	IL8 signalling
IFITM1	Interferon induced transmembrane protein 1	2.13E-03	2.375	IFN signalling
CCR3	Chemokine (C-C motif) receptor 3 Gene	1.31E-03	1.655	CCR3 signalling in eosinophils
				Chemokine signalling
ACTA2	Actin, alpha 2, smooth muscle, aorta	1.77E-03	1.640	Crosstalk between DC and NK cells
				Leukocyte extravasation signalling
				Integrin signalling
TAB1	Mitogen-activated protein kinase 7 interacting protein 1	2.82E-03	1.276	iNOS signalling
				NF-kB signalling
				IL10 signalling
				Toll-like receptor signalling
CD40LG	CD40 ligand	1.41E-03	−1.740	T helper cell differentiation
				CD40 signalling
				IL12 signalling and production in macrophages
				NF-kB signalling
JUN	Jun oncogene	4.08E-03	−1.587	CD28 signalling in T helper cells
				IL12 signalling and production in macrophages
				Production of nitric oxide and reactive oxygen species
				T-cell receptor signalling
				iNOS signalling
				IL8 signalling
				IL10 signalling
PLCG1	phospholipase C, gamma 1	5.33E-03	−1.541	iCOS-iCOSL signalling in T-helper cells
				PCK signalling in T-lymphocytes
				Production of nitric oxide and reactive oxygen species
				Phospholipase C signalling
				IL15 signalling
PRKCQ	protein kinase C, theta	3.14E-03	−1.461	T-cell receptor signalling
				CCR5 signalling in macrophages
				Natural killer cells signalling
				Apoptosis signalling
				B-cell receptor signalling
PRKD3	protein kinase D3	6.26E-03	−1.440	Calcium-induced T-lymphocyte apoptosis
				LPS stimulated MAPK signalling
				CCR5 signalling in macrophages
				CCR3 signalling in eosinophils
				CXCR4 signalling

The genes analysed here were used when comparing non-immunised mice to mice immunised with a combination of T-cell-epitope-containing peptides (T14 + T15 + T16)

### Biological processes

**Fig. 3:**
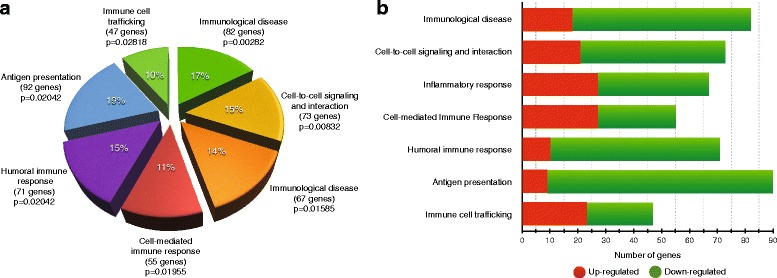
The biological process involved in spleen differentially expressed genes compared to the non-immunised control group. **a** The total number of genes is showed in parentheses and the significance of each biological process is indicated by its *p* value. **b** The amount of both up- and down- differentially expressed genes is indicated for each biological process. *Red* represents the amount of up-regulated genes whilst *green* represents down-regulated ones

The differentially expressed genes were also analysed to identify changes in the biological process associated with the immune response, taking the amount of genes being differentially expressed into account (*p* < 0.05) and each biological process’ statistical significance, regardless the fold change value. Figure 3 shows that the most significant biological processes changed by immunisation with our vaccine candidate were related to the induced- immune response. The top three functions associated with an immune response were immunological disease (including up-regulation of C3, MMP9, BRCA2, CCRR, CXCR2, ALOX5), cell-to-cell signalling and interaction (including up-regulation of ANXA1, C3, CLIC4, S100A9, ELANE) and inflammatory response (including up-regulation of FPR1, C1QA, C5AR1, CD200R1, LTBR).

**Fig. 4:**
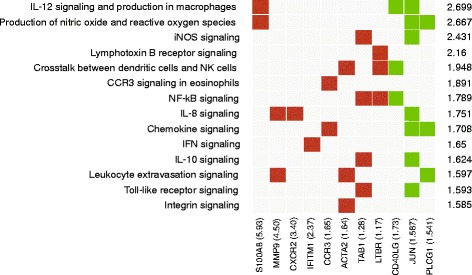
The most representative signalling pathways and their associated up- and down-regulated genes are represented. The fold change of each gene is shown in parenthesis. The significance (−log *p* value) of each signalling pathway is also indicated by the scale to the right of the graph. Signalling pathway analysis was done only in the group of mice immunised with the combination of peptides containing T-cell epitopes (T14, T15, T16). *Red* and *green squares* represent genes belonging to each signalling pathway, whereas *gray squares* indicate absence of those genes in that pathway

It is worth stressing that down-regulation was observed in most differentially expressed genes (134); however, the fold change values were higher in the up-regulated genes. Differential expression of the most significant genes was ranked as follows: C3 > ANXA1 > CXCR2 > LTBR > MMP9 > ALOX5 > C5AR1 > S100A9 > ELANE > CCR3. Figure 4 shows their participation in different biological functions, whilst a detailed list of all differentially expressed genes and their participation in functions and associated-functions is given (Additional file 5: Table S4). Our approach focused on genes being up-regulated and their canonical pathways as our goal was to find a link between an immune response induced upon vaccination and the protective efficacy so achieved; however, it is worth mentioning that most genes being differentially expressed belonged to the down-regulated group and affected a wide range of canonical and signalling pathways. CD28 signalling and iCOS-iCOSL in T-helper cells and T-cell receptor signalling were the most representative canonical pathways being down-regulated. Concerning genes, CD40LG, JUN1, PLCG1 and IKBKE had the highest down-regulation in the aforementioned pathways.

### PCR for microarray validation

Semi-quantitative reverse-transcription PCR was used in the present study for validating differentially expressed genes, using free ImageJ software [41] for calculating each band’s intensity; six genes were randomly selected for such confirmation.

**Fig. 5:**
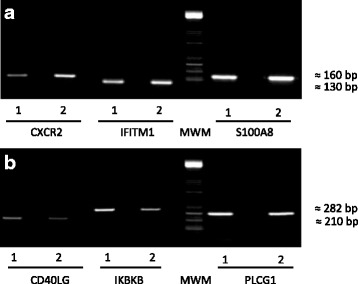
The microarrays were validated by using PCR reactions. Panel **a** depicts up-regulated genes and panel **b** down-regulated genes. *Lane 1* represents the corresponding PCR amplification of the indicated gene from untreated mice and *lane 2* represents the corresponding PCR amplification of the indicated gene from the immunised mouse group. Each mouse’s RNA was pooled and used for RT-PCR and the genes for validation were randomly assigned. The results are representative of three individual experiments. MWM: Molecular Weight Marker

The PCR expression patterns agreed with those obtained in the microarrays. Figure 5 shows that all genes being up-regulated had higher band intensity in the group of mice immunised with the vaccine candidate compared to the untreated group, whereas down-regulated genes had lower band intensity in the immunised group. Fold change was calculated by measuring each band’s intensity: CXCR2 2.70, IFITM1 1.40, S100A8 1.23, CD40LG -1.86, IKBKB -1.57 and PLCG1 -1.18.

## Discussion

The present work involved using a microarray-based technology together with protection studies against *F. hepatica* to better understand the gene expression profile and immunological mechanisms leading to protection in mice immunised with a novel anti-*F. hepatica* vaccine candidate.

*F. hepatica* cathepsin B and amoebapore-like proteins were selected as vaccine candidates in the present study. According to the pertinent literature, it is well known that *F. hepatica* cathepsins are secreted proteins which are differentially expressed during the life stages of flukes; they have different associated functions in each stage and are also considered significant vaccine candidates. *F. hepatica* cathepsin B is mainly expressed in metacercariae and newly excysted juveniles whilst cathepsin L is mainly expressed in newly excysted juveniles and adult worms [45]. *F. hepatica* cathepsins are considered important vaccine candidates; several studies have demonstrated their protection-inducing ability in different experimental models [46–49]. *F. hepatica* amoebapore proteins have shown amino acid sequence similarity with saposin-like proteins. These proteins have been shown to be highly immunogenic and their inducing-protection ability has also been demonstrated in mice and rabbits. Concerning *F. hepatica* saposin-like protein expression, up-regulation has been demonstrated in eggs, newly excysted juveniles and adults and down-regulation in miracidia [50]. The recently published draft genome for *F. hepatica* identified three proteins from amoebapore-like proteins referred to as saposin-like proteins. The gene expression profile has revealed up-regulation of the amoebapore-like protein (putative, uncharacterised; Uniprot blast hits Q4KSL8; Q24939; Q24938; Q9NAT2; B6ZBP3) in newly excysted juveniles, juvenile (21 days) and adult stages. The amoebapore-like protein (Uniprot blast hits Q9NAT2; B6ZBP3) is down-regulated in the newly excysted juveniles stage but up-regulated in the juvenile (21 days) and adult stages. The third amoebapore-like protein (Uniprot blast hits Q9NAT2; B6ZBP3; Q4KSL7; Q4KSL8; Q24939) is also down-regulated in newly excysted juveniles stages but highly up-regulated in the juvenile (21 days) and adult stages [51].

To date, little is known concerning the development of subunit-based, chemically synthesised anti-*F. hepatica* vaccines [52, 53]. The present study involved selecting mouse survival rates (represented by Kaplan-Meier survival curves) and hepatic damage score as indicators for protection. Recovered worm count should also be included as protection indicator in an ideal scenario; however, the number of worms recovered per animal was too low when using the mouse model of infection for *F. hepatica* experiments; drawing conclusions for protection based on worm count could thus be biased. It was also considered that worm count in infected mice was indeed a less reliable method for assessing protection. Mice immunised with a combination of synthetic peptides containing either B- or T-cellepitopes increased mouse survival rate in the present study. Despite the peptides tested were originally designed based on their good predicted binding to the H2-Ed MHC type displayed by the syngeneic BALB/c mice as a model to obtain a specific response against *F. hepatica*, testing the protective immune response induced in an outbred model such as CD1 mice could represent better the variability observed in the target population for vaccination such as sheep and cattle.

The combination of peptides containing T-epitopes (T14 + T15 + T16; group 6) induced the highest survival rate, thereby supporting our hypothesis that a multi-epitope-based vaccine is necessary for obtaining higher immune-protective responses against helminthic infection. A previous study by our group demonstrated the immunoprotective efficacy of single peptides containing B- or T-cell epitopes against experimental infection with *F. hepatica* metacercariae, reaching good immunoprotective levels with the so-called peptide T15 [29]. Here, we have demonstrated that adding peptides T14 and T16 induced better protective levels than the single peptide T15, taking the Kaplan-Meier survival curves and hepatic damage into account.

Adding peptides containing B-cell epitopes to our vaccine candidate induced a loss of immunogenicity as measured by determining IgG levels and immunoprotection levels became reduced. It is worth noting that vaccinating mice with ADAD vaccination system components induced little protection, as indicated by Kaplan-Meier survival curves, and little reduction was observed regarding hepatic damage.

A microarray-based methodology was used to identify the gene expression profile in BALB/c mice immunised with our most protective antigen combination formulated in ADAD vaccination system (Qs + AA0029 + T14 + T15 + T16) and the uninfected control mice to gain fresh insight into the molecular and immunological basis leading to protection. Eosinophilia has been associated with helminth infection, although its precise role in conferring protection is still controversial and several studies have been concerned with the role of eosinophils in protection by using both in vitro and in vivo studies [54–56]. CCR3 is a receptor for C-C type chemokines, including eotaxin, MCP-3, MCP-4 and RANTES, and is highly expressed in eosinophils. Our study found up-regulation of the CCR3 chemokine receptor in mice immunised with our vaccine candidate, thus supporting the hypothesis that eosinophils could be mediating mechanisms leading to protection in *F. hepatica* infection. By contrast, other studies have shown that eosinophils suffer induced-apoptosis by excretory/secretory products from *F. hepatica*, thus suggesting that proposing a role for such cells in inducing protection should be taken with caution [57]. IL-8 receptor gene (CXCR2) up-regulation was also found. CXCR2 is mainly expressed on neutrophil surface, providing some information concerning the possible mechanisms involved in Th2 cell-mediated immune clearance pathways. Up-regulation of other types of C-X-C family chemokines (i.e. CXCR4) has been shown to be essential in animals which are genetically-resistant to nematode parasites [58].

Up-regulation of the endogenous toll-like receptor-4 agonist was also achieved by the immunisation trial through expression of the S100A8/S100A9 complex, known as calprotectin. These two members of the damage associated molecular pattern family are secreted during phagocyte stress response [59]. Evidence has been presented regarding neutrophil and S100A8 neutrophil chemokine localisation in fibrotic areas in a murine model of *Schistosoma japonicum*, suggesting its implication in the induction of fibrosis [60]. S100A8 and S100A9 are up-regulated in liver and spleen during *S. japonicum* infection. It has also been suggested that S100A8 up-regulation may protect against oxidative tissue damage. There is no evidence to date regarding the precise role of the S100A8/S100A9 complex concerning *F. hepatica* infection or acquiring protective immunity. The present study’s results suggested activation by innate immunity with up-regulation of both S100A8 and S100A9 in the spleen of mice immunised with our vaccine candidate, a key factor in inducing protective immunity, at least in a murine model.

Reports in the pertinent literature have investigated the role of C3 during schistosome infection in a murine model. C3 depletion has led to a significant reduction in Th2-associated cytokines without any correlation with worm development or liver pathology. C3-defficient mice have not been able to effectively clear adult worms after treatment with praziquantel [61]. C3 depletion seems to be agree as complement activation do not represent an immunologic mechanism leading to immunoprotection against *F. hepatica* infection. Also, nitric oxide is related to Th1 immune response with subsequent macrophage activation, unleashing nitric oxide liberation as an immunoprotector-associated mechanism. However, we could not provide any experimental evidence showing its correlation with hepatic damage or fluke burden reduction.

It has also been shown that nitric oxide production is enhanced by the synergic effect of both IL-12 and IL-8. IL-12 is considered to be a cytokine inducing a Th1 immune response, with IFN-γ overproduction. IL-12 plays a key role in helminthic infection by inhibiting Th2 immune responses which are essential for a parasite surviving inside a mammalian host [62]. It was observed in our study that the IL-12 signalling pathway was significantly altered by the immunisation trial, being the genes S100A8, CD40LG and JUN differentially expressed, making it a key factor leading to protection. Significant alteration in IL-8 signalling was observed after mice were immunised with our vaccine candidate, involving up-regulation of receptors for IL-8, MMP9 and CXCR2. Nitric oxide production has been studied in vitro in infection caused by helminths involving *Trichinella spp, Dirofilaria immitis* and *Ascaris suum* antigens; a correlation has been found between antigen-dependent stimulation and nitric oxide levels [63–65]. Nitric oxide could regulate inflammation produced by egg release, prevent hepatic cell damage, the spread of damage in the liver and reduce granuloma formation by *S. mansoni* [66]. Concerning *F. hepatica* infection, nitric oxide production has been studied in infected rats, showing reduced nitric oxide levels 7 and 14 days p.i. Such reduction has been associated with excretory/secretory released antigens, constituting one of the defence mechanisms used by this parasite during its migration through the peritoneal cavity of a mammalian host [67]. It could be hypothesised that changes in nitric oxide signalling pathway induced by the immunisation trial could have been involved in inducing protective immunity, thereby becoming a necessary condition for such immunoprotection. No previous reports are available concerning the expression levels of MMP9 during *F. hepatica* infection. In *S. mansoni*-infected mice there is evidence of changes in MMP gene family expression in the chronic phase of the disease, which is associated to degradation of collagen deposited in tissues. Up regulation of MMP2, MMP3 and MMP8 has been reported in *S. mansoni*-infected mice but no significant differential expression was observed in MMP9 gene [68]. *S. mansoni*-infected patients have had low CXCR1 and CXCR2 frequency during the acute phase of the disease but high IL-8 levels in sera [69]. Besides high CXCR1 and CXCR2 expression in our study, we could not provide any evidence of IL-8 in conferring protective immunity; however, it provided a first insight concerning its implication in *F. hepatica* protection-inducing mechanisms.

## Conclusions

The present study’s findings have highlighted the immunoprophylactic potential of T-cell-epitope-containing synthetic peptides when used in combination and formulated in the ADAD vaccination system, thus supporting the idea that developing multi-epitope, subunit-based and chemically-synthesised vaccines produces better results than immunisation with single antigens. We have also reported for the first time a transcriptional profile concerning the splenocytes of mice immunised with a promising anti-*F. hepatica* vaccine candidate. The results showed that many molecular mechanisms and signalling pathway alterations are potentially involved in conferring protective immunity, at least in a murine model. We have highlighted the potential involvement of genes and signalling pathways associated with nitric oxide production, IL-12 signalling and IL-8 signalling in conferring protective immunity. This study has provided us with insights regarding possible cellular and molecular mechanisms involved in protection against *F. hepatica*; however further studies are necessary for experimentally confirming our hypothesis.

## Additional files

Additional file 1: Table S1.
**a** The amino acid sequences for each peptide selected for the in vivo protection studies. The GenBank accession numbers for the entire protein from which the peptides were derived and the induced immune responses are also shown. **b** Nucleotide sequence for PCR amplification of genes being up-regulated and down-regulated following the immunisation trial. (DOCX 78 kb)
Additional file 2: Figure S1. The antibody levels (IgG) in mice immunised with the synthetic peptides. An ELISA was performed to assess the success of the immunisation procedure. Sera samples were obtained two weeks after the last immunisation. (EPS 126 kb)
Additional file 3: Figure S2. Agilent 2100 bioanalyzer electropherograms showing individual mouse spleen RNA samples obtained two-weeks after the last immunisation. Panels A, B and C represent RNA samples from untreated mice and panels D, E and F represent RNA samples from mice immunised with the combination of peptides containing T-cell epitopes (T14 + T15 + T16). (EPS 1277 kb)
Additional file 4: Figure S3. Box plot showing the normalised unscaled standard error (NUSE) for each microarray. The non-immunised control group is shown in red whilst the immunised group (T14 + T15 + T16) is shown in blue. (EPS 184 kb)
Additional file 5: Table S2. The table lists the number of genes differentially expressed as a direct result of the immunisation trial. Differential expression has been calculated in each microarray by using the robust microarray analysis algorithm and the significance analysis of microarrays method. Up-regulated genes are shown in red whilst down-regulated are shown in green. **Table S3.** The Ingenuity Pathway Analysis tool was used for identifying the signalling pathways and their associated-genes differentially expressed by the immunisation trial. Up-regulation is shown in red whilst down-regulation is shown in green. **Table S4.** Biological functions and their associated genes differentially expressed by the immunisation trial were identified by using the Ingenuity Pathway Analysis tool. Up-regulation is shown in red whilst down-regulation is shown in green. **Table S5.** Immunisation of mice with a combination of peptides containing T-cell epitopes led to the differential expression of 37 genes (*p* <0.05; fold change +/− 2). Up-regulation is shown in red whilst down-regulation is shown in green. (XLSX 257 kb)

## Abbreviations

ADAD: Adjuvant adaptation
ALOX5: Arachidonate 5-lipoxygenase
ANOVA: Analysis of variance
ANXA1: Annexin A1
BRCA2: Breast cancer type 2
C1QA: Complement C1q A chain
C3: Complement C3
C5AR1: Complement C5a receptor 1
CD200R1: CD200 receptor 1
CD40LG: CD40 ligand
CLIC4: Chloride intracellular channel 4
CXCR2: Chemokine receptor 2
ELANE: Elastase neutrophil expressed
ELISA: Enzyme-linked immunosorbent assay
FPR1: Formyl peptide receptor 1
IFITM1: Interferon induced transmembrane protein 1
IFN: Interferon
IKBKE: Inhibitor of kappa light polypeptide gene enhancer in B cells, kinase epsilon
IL: Interleukin
JUN1: Jun proto-oncogene 1
LTBR: Lymphotoxin beta receptor
MMP9: Matrix metalloprotease 9
PCR: Polymerase chain reaction
PKC: Protein kinase C
PLCG1: Phospholipase C gamma 1
RNA: Ribonucleic acid
S1008A: S100 calcium-binding protein A8
TGF: Transforming growth factor
TMHMM: Transmembrane helix prediction using hidden Markov model
