# Supplementary material for: Transcriptome profiling of gene expression during immunisation trial against Fasciola hepatica: identification of genes and pathways involved in conferring immunoprotection in a murine model
Source: BMC Infect Dis. 2017 Jan 23;17:94. doi: 10.1186/s12879-017-2205-3 (PMC5259852; doi:10.1186/s12879-017-2205-3)
Supplement: Additional file 1: Table S1. — a The amino acid sequences for each peptide selected for the in vivo protection studies. The GenBank accession numbers for the entire protein from which the peptides were derived and the induced immune responses are also shown. b Nucleotide sequence for PCR amplification of genes being up-regulated and down-regulated following the immunisation trial. (DOCX 78 kb) [file 12879_2017_2205_MOESM1_ESM.docx]

**Additional file 1: Table S1. a** The amino acid sequences of each peptide selected for the *in vivo* protection studies.

| GenBank | Description | | Protein  length (aa) | Synthesised peptide  sequence / Epitope | Position | Immune response |
| --- | --- | --- | --- | --- | --- | --- |
| AAB02579.1 | | Amoebapore homologue | 102 | KGAGSSQDACIKFIQYEVDG / B1 | 63-82 | Th1/Th2 |
| AAB02579.1 | | Amoebapore homologue | 102 | KGAGSSQDATIKFIQYEVDG / B2 | 63-82 | Th10 |
| ABU62951.1 | | Cathepsin B | 337 | ISEIRDQSSTSSTWAVSSAS / B5 | 102-121 | Th1/Th2/Th10/Th17 |
| ABU62951.1 | | Cathepsin B | 337 | GVENGVKYWLIANSWNEGWG / B6 | 293-312 | Th2 |
| AAB02579.1 | | Amoebapore homologue | 102 | TVNLVKRLLQNSVVE / T14 | 37-51 | Th2 |
| AAF88069.1 | | Amoebapore-like protein | 101 | DYIIDHVDQHNATEI / T15 | 80-94 | Th1 |
| ABU62925.1 | | Cathepsin B | 337 | DRNTQRQTVRYSVSE / T16 | 69-83 | Th1/Th2 |

GenBank accession number from the entire protein the peptides belonged to and the induced immune response is also shown.

**Additional file 1: Table S1. b** Nucleotide sequence for PCR amplification of genes being *up-regulated* and *down-regulated* after immunisation trial.

| Gene symbol | Ensembl code | Primer sequence Forward (5’ 🡪 3’) / Reverse 3’🡪5’ | Primer length  (pb) | Tm  (ºC) | Product length  (pb) |
| --- | --- | --- | --- | --- | --- |
| CXCR2 | ENSMUSG00000026180 | ACTCCTTGGTGATGCTGGTC  ACAGGGTTGAGCCAAAAGT | 20 | 70.3  68.3 | 160 |
| IFITM1 | ENSMUSG00000025291 | GGTGGTTGTACTGGGGTCAC  CCAGGCAGCAGAAGTTCATG | 20 | 72.3  70.3 | 134 |
| S100A8 | ENSMUSG00000056054 | ATGCCGTCTGAACTGGAGAA  TGCACAAACTGAGGACACTC | 20 | 68.2  68.2 | 140 |
| CD40LG | ENSMUSG00000031132 | CAGTGGGCCAAGAAAGGATA  GGTATTTGCCGCCTTGAGTA | 20 | 68.2  68.2 | 219 |
| IKBKE | ENSMUSG00000042349 | TGTACAAGGCCCGAAACAAG  CGCAGCACCACTAGGAACT | 20 | 70.3  72.3 | 226 |
| PLCG1 | ENSMUSG00000016933 | GACAGCCTGGTTGACCTCAT  CTCTCTGGGCCTTGTAGTCG | 20 | 68.2  69.7 | 282 |
